# A SNP associated with alternative splicing of *RPT5b *causes unequal redundancy between *RPT5a *and *RPT5b *among *Arabidopsis thaliana *natural variation

**DOI:** 10.1186/1471-2229-10-158

**Published:** 2010-08-03

**Authors:** Anouchka Guyon-Debast, Alain Lécureuil, Sandrine Bonhomme, Philippe Guerche, Jean-Luc Gallois

**Affiliations:** 1 Institut Jean-Pierre Bourgin, UMR1318 INRA-AgroParisTech, Versailles, France, F-78000 Versailles, France; 2 Institut National de la Recherche Agronomique-UR1052 Station de Génétique et d'Amélioration des Fruits et Légumes, Domaine Saint Maurice, BP94, F84143, Montfavet, France

## Abstract

**Background:**

The proteasome subunit RPT5, which is essential for gametophyte development, is encoded by two genes in *Arabidopsis thaliana*; *RPT5a *and *RPT5b*. We showed previously that *RPT5a *and *RPT5b *are fully redundant in the Columbia (Col-0) accession, whereas in the Wassilewskia accession (Ws-4), *RPT5b *does not complement the effect of a strong *rpt5a *mutation in the male gametophyte, and only partially complements *rpt5a *mutation in the sporophyte. *RPT5b^Col-0 ^*and *RPT5b^Ws-4 ^*differ by only two SNPs, one located in the promoter and the other in the seventh intron of the gene.

**Results:**

By exploiting natural variation at *RPT5b *we determined that the SNP located in *RPT5b *intron seven, rather than the promoter SNP, is the sole basis of this lack of redundancy. In Ws-4 this SNP is predicted to create a new splicing branchpoint sequence that induces a partial mis-splicing of the pre-mRNA, leading to the introduction of a Premature Termination Codon. We characterized 5 accessions carrying this A-to-T substitution in intron seven and observed a complete correlation between this SNP and both a 10 to 20% level of the *RPT5b *pre-mRNA mis-splicing and the lack of ability to complement an *rpt5a *mutant phenotype.

**Conclusion:**

The accession-dependent unequal redundancy between *RPT5a *and *RPT5b *genes illustrates an example of evolutionary drifting between duplicated genes through alternative splicing.

## Background

Gene duplications contribute to evolution [1]. They arose mostly through polyploidy (i.e. genome duplication) that has been a widespread phenomenon among Angiosperms [2]. *Arabidopsis thaliana *has undergone several rounds of genome duplication and up to 23% of its proteome is encoded by duplicated genes [3]. The evolutionary outcomes of gene duplication were first theorized by Ohno [4], who predicted that although duplicated genes are redundant at first, they soon evolve and one of the duplicates either accumulates detrimental mutations and is lost, or gains new function(s). As a consequence, over time the duplicated genes become partially or no longer redundant. In some cases, both genes may have retained unequal redundancy: a mutation in one of the two duplicated genes causes a mutant phenotype while a mutation in the other does not; in the double mutant, this phenotype is strongly enhanced [5] At its utmost, independent evolution of duplicated genes among close accessions is the basis of incompatibility known as the Dobzhansky-Muller type syndrome [6,7].

Gene evolution can be assessed by studying natural variation in a single species. A recent survey highlighted that nucleotide sequence polymorphism appears in all gene regions, both coding and non-coding [8]. In particular, alternative splicing, that creates multiple mRNA from the same gene unit, has been described recently as a source of variation among natural accessions. For example, this process alters *FLC *function in the Arabidopsis accession Bur-0 [9] as well as in some *Brassica rapa *accessions [10]. Although evolution through alternative splicing may contribute to the creation of new functions through exon sliding [11], it has mainly been described as inducing Premature Termination Codon (PTC). Transcripts affected by PTC are often targets of Nonsense-Mediated Decay (NMD), leading to their degradation [12,13]. Interestingly, it has been shown that alternative splicing is often conserved between species as diverse as Rice and *Arabidopsis*, suggesting that this process has an important role in gene function [14]. However, the extent of variation in alternative splicing among accessions in a single species is not known.

In previous work we showed that two *Arabidopsis *genes, *RPT5a *and *RPT5b*, are necessary for the development of both gametophytes [15]. These genes encode an AAA-ATPase subunit that acts as a component of the 26S proteasome machinery. Loss-of-function for both genes is male and female gametophyte lethal. In the Col-0 accession, both *RPT5a *and *RPT5b *genes act in a redundant manner. However, in the Ws-4 accession, *RPT5b *does not complement for *rpt5a *mutant phenotype in male gametophyte (and to a lesser extent in the sporophyte). Sequencing the 4800 bp *RPT5b *locus identified only two SNPs that differed between the *RPT5b^Col-0 ^*and *RPT5b^Ws-4 ^*genes, one located in the promoter (named SNP1) and the other at the end of the seventh intron (named SNP2). Those polymorphisms will be referred to as SNP1^Ws ^(or SNP1^Col^) and SNP2^Ws ^(or SNP2^Col^). These findings led us to suggest that inactivation of the *RPT5b^Ws-4 ^*allele could be a result of either diminished *RPT5b *expression related to SNP1^Ws^, or else due to a mis-splicing event occurring at intron 7 related to SNP2^Ws ^[15]. Here we address this question by looking among 487 Arabidopsis accessions for *RPT5b *alleles with polymorphisms similar to those detected in the Ws-4 accession. Redundancy between *RPT5a *and *RPT5b *genes was then tested regarding each of these two SNPs. This approach allowed us to demonstrate that SNP2 is responsible for *RPT5b *inactivation through pre-mRNA mis-splicing, leading to PTC. This A-to-T variation in intron 7, which is shared by 5 unrelated accessions, is predicted to create a new branchpoint sequence upstream from *RPT5b *intron seven 3' splice site, leading to its mis-splicing.

## Results

### Screening of Arabidopsis accessions for variation at the *RPT5b *locus

Our previous work focussed on two accessions for which *Arabidopsis *insertional mutant collections are available, Col-0 and Ws-4. We presumed that the two SNPs highlighted between the *RPT5b*^*Col-0 *^and *RPT5b*^*Ws-4 *^alleles were responsible for their different abilities to complement the male gametophyte lethal phenotype in a *rpt5a *background.

We previously showed that a strong *rpt5a-1 *male gametophyte mutation was complemented by a transgene harbouring a 4 kb *RPT5b*^*Col *^construct [15]. Likewise, 3 constructs were transformed into *rpt5a-1/RPT5a *plants, each harbouring the same 4 kb of *RPT5b *with different combinations of Col/Ws SNP1 and SNP2. It was tested whether those constructs would allow the transmission of *rpt5a-1 *through the male gametophyte (Additional file 1: Figure S1). Surprisingly, all 4 *RPT5b *constructs (SNP1^Col ^SNP2^Col^, SNP1^Ws ^SNP2^Ws^, SNP1^Col ^SNP2^Ws ^and SNP1^Ws ^SNP2^Col^) did complement the *rpt5a-1 *mutation. The empty binary vector used as a control did not. It has to be noted that those transformed plants expressed the endogenous *RPT5b*^*Ws *^gene as well as the *RPT5b *transgene. It is very likely that the additive expression of the endogenous *RPT5b *together with the transgene is sufficient to complement the *rpt5a-1 *mutation and is therefore masking the partial loss of function of the *RPT5b*^*Ws *^allele.

**Figure 1:**
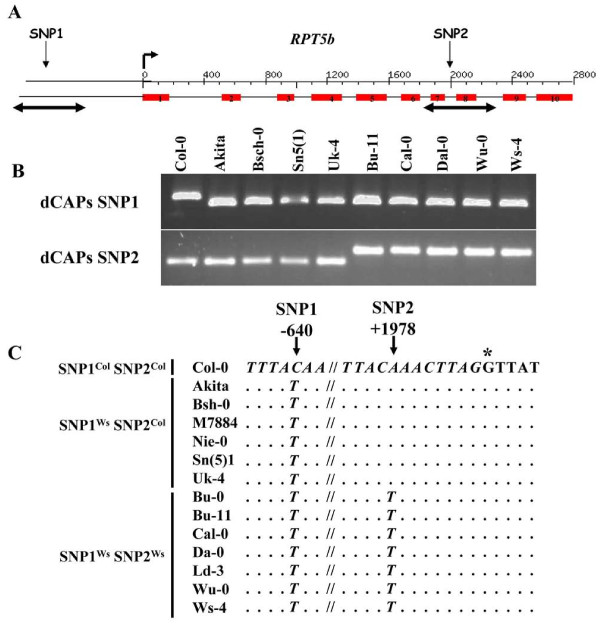
**Screening for *RPT5b *polymorphism among Arabidopsis natural variation**. **A**, Graphic representation of *RPT5b*. Red boxes represent exons. The start of translation is indicated by a broken arrow. Both promoter and intron seven SNPs (respectively SNP1 and SNP2) are indicated. Double-end arrows located below *RPT5b *cover the 500 bp fragments that have been re-sequenced. **B**, Two dCAPS markers were used to discriminate SNP1 and SNP2 between Col and Ws accessions (see Methods). Results are also shown for the 8 selected accessions that show a non-Col signature for SNP1 and/or SNP2. C, Display of sequences around SNP1 (in promoter) and SNP2 (in intron 7) following partial *RPT5b *re-sequencing. The Col sequence is given as reference. Non-coding sequences are italicised while coding sequence (beginning of exon 8) are not. The first base of exon 8 is indicated by a star.

As a complementary approach, we looked for additional accessions displaying polymorphism at either the promoter (SNP1) or within intron 7 (SNP2) using previously designed dCAPS markers ([15] and Fig. 1A and 1B). 487 accessions from the Versailles Resource Centre were screened for both dCAPS (Fig. 1B and not shown). Both Col plants (Col-0 and Col-7) contained in the set of accessions displayed a Col/Col digestion profile (dCAPS SNP1/dCAPS SNP2) as well as the majority (468) of tested accessions. All five Ws accessions (Ws, Ws-1, Ws-2, Ws-3 and Ws-4) as well as 6 new accessions were found to display a Ws/Ws profile and 6 new accessions showed a Ws/Col profile. No accession that would present a (Col dCAPS SNP1/Ws dCAPS SNP2) profile was found. To confirm the first screening, 12 accessions were chosen (Table 1) and were grown for new genomic DNA extraction. Two 500 bp regions, each one encompassing one SNP, were sequenced to confirm the initial dCAPS screening (Fig. 1A). This was of particular importance as the dCAPS could only discriminate between non-Ws and Ws status (SNP1) and non-Col and Col status for SNP2. All sequence data showed that among the accessions chosen there were no variations other than those we previously described between Col-0 and Ws-4 (Fig. 1C). The identification of accessions with different haplotypes gives us the opportunity to test which SNP is responsible for the observed phenotype.

**Table 1:** List of Arabidopsis accessions selected for this study

Name	VNAT reference number	Country of origin
**Ws-4**	**530AV**	Belarus
**Da-0**	**126AV**	Germany
**Bu-0**	**182AV**	Germany
**Bu-11**	**464AV**	Germany
**Wu-0**	**246AV**	Germany
**Ld-3**	**212AV**	Unknown
**Cal-0**	**184AV**	United Kingdom
**Akita**	**252AV**	Japan
**Bsch-0**	**125AV**	Germany
**Nie-0**	**139AV**	Germany
**M7884S**	**227AV**	Unknown
**Sn(5)1**	**237AV**	Czechoslovakia
**Uk-4**	**522AV**	Germany
**Col-0**	**186AV**	Poland

More informations can be obtained at http://dbsgap.versailles.inra.fr/vnat

For practical matter relative to plant crosses, we retained 4 accessions for each haplotype displaying about the same flowering time as Col-0 and Ws-4. Those selected accessions are: Bu-11, Cal-0, Da-0 and Wu-0 for the SNP1^Ws^/SNP2^Ws ^haplotype, and Akita, Bsch-0, Sn5(1) and Uk-4 for the SNP1^Ws^/SNP2^Col ^haplotype.

### The lack of complementation of *rpt5a *mutant phenotype by *RPT5b *gene correlates with SNP2^Ws ^but not with SNP1^Ws^

**Figure 2:**
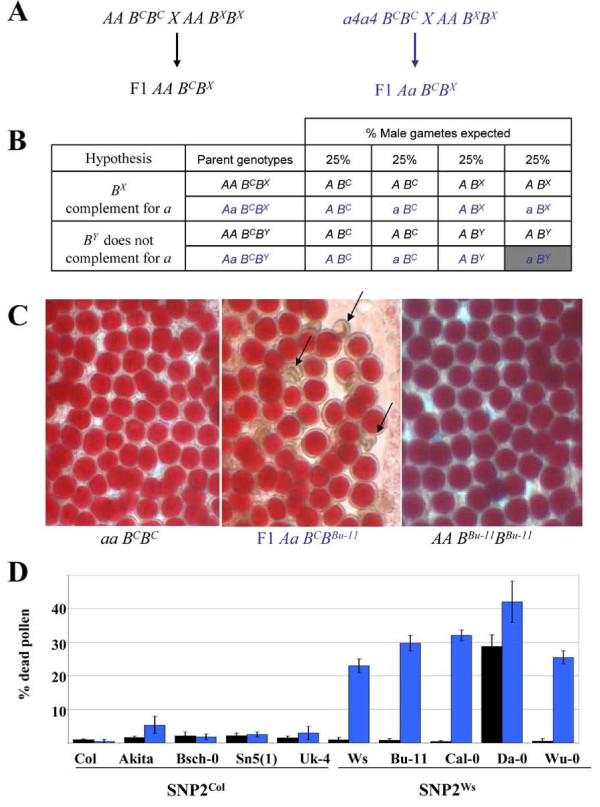
**A SNP in *RPT5b *intron 7 correlates with unequal redundancy between *RPT5a *and *RPT5b *in pollen development**. **A**, Schematic representation of the F1 crosses used in this study. A and a represent wild-type *RPT5 *and *rpt5a-4 *mutated allele, respectively. *B*^*C *^represents the Col allele *RPT5b *while X stands here for a non-Col accession. **B**, Gamete segregations for the F1 plants obtained in **A**. Two hypotheses are taken into consideration: that either the non-Col *RPT5b *allele complements the *rpt5a *mutation (*B*^*X*^, from the X accession) or that it does not (*B*^*Y *^from the Y accession). For each genotype, the percentages of expected gametes are indicated. Hence if the hypothesis is correct the percentage of dead pollen is either null or indicated by the grey column. **C**, Mature pollen stained with Alexander's stain in anther locules from a *Aa4 B*^*C *^*B*^*Bu-11 *^F1 hybrid and parents. Only the F1 plants show some dead pollen (indicated by arrows). **D**, Percentage of dead pollen determined by Alexander staining and subsequent counting in F1 *AA B*^*C*^*B*^*X *^(black boxes) and *Aa4 B*^*C*^*B*^*X *^(blue boxes where X stands for the accession written below the graph) plants. For each genotype, 300 grains were counted per plant. Values are shown as means on 4 independent plants (±SD).

We previously showed that the Col-0 allele of *RPT5b *(*RPT5b^Col-0^*) rescued the male gametophyte defect due to *rpt5a *loss of function whereas *RPT5b^Ws-4 ^*did not, showing that the *RPT5b^Ws-4 ^*allele is not functional in the male gametophyte. Male gametophytes harbouring an *rpt5a *; *RPT5b^Ws-4 ^*genotype degenerate as shown by the vital marker Alexander staining [15]. Pollen phenotype is readily checked on the F1 progeny from crosses between the *rpt5a-4 *mutant (in a Col-0 background) and any other accession: 100% of the pollen should be viable if the *rpt5a-4 *mutation is complemented -by *RPT5b*^*x*^- but only 75% will be viable if there is no complementation (Fig. 2A and 2B). Each selected accession was crossed to wild-type Col-0 to assess any incompatibility between accessions that is unrelated to RPT5. At the same time, each selected accession was crossed to homozygous *rpt5a-4 *mutants (*rpt5a-4/rpt5a-4; RPT5b*^*Col-0*^/*RPT5b*^*Col-0*^) to test whether the *rpt5a-4 *mutation could be rescued by the various *RPT5b *alleles. Ws-4 and Col-0 plants were used as controls. F1 plants were grown and pollen viability was tested (Fig. 2C and 2D). For 7 out of the 8 tested accessions, no pollen incompatibility was observed: consequently the cross with *rpt5a-4 *mutant could be used to test the (lack of) complementation of the *rpt5a *mutation. Only one accession, Da-0 (SNP1^Ws^/SNP2^Ws ^haplotype), was found to trigger incompatibility when crossed to Col-0. F1 Da-0 × Col-0 showed a 28% pollen lethality that would be consistent with 2 independent genes leading to pollen lethality and could correspond to another example of Dobzhansky-Muller type incompatibility [6].

F1 progenies from crosses of *rpt5a-4 *mutant with Akita, Bsch-0, Sn5(1) and Uk-4 were found to display almost no dead pollen (Fig. 2D). This means that *RPT5b*^*Akita*^, *RPT5b*^*Bsch-0*^, *RPT5b*^*Sn5(1)*^, and *RPT5b*^*Uk-4 *^alleles complement the *rpt5a-4 *mutation, in contrast to *RPT5b*^*Ws-4*^. As these accessions have a SNP1^Ws^/SNP2^Col ^haplotype, it can be concluded that the SNP1^Ws ^is not involved in the process that impairs the *RPT5b*^*Ws-4 *^allele from complementing the *rpt5a-4 *mutation. This is consistent with previous data showing that the promoter SNP1 did not seem to modify the GUS gene expression pattern when a 1200 bp promoter, either from Col-0 or from Ws-4, was fused to the GUS gene reporter [15].

By contrast, in F1 progeny from crosses of *rpt5a-4 *mutant with Bu-11, Cal-0 or Wu-0, approximately 25% non-viable pollen was observed, as for *rpt5a-4/RPT5a; RPT5b*^*Col-0*^/*RPT5b*^*Ws-4 *^plants (Fig. 2C and 2D). This pollen lethality shows that *RPT5b*^*Bu-11*^, *RPT5b*^*Cal-O *^and *RPT5b*^*Wu-*0 ^alleles do not complement the *rpt5a-4 *mutation. As these accessions have a SNP1^Ws^/SNP2^Ws ^haplotype, and because SNP1 was shown not to be involved, we conclude that the SNP2^Ws ^is responsible for the lack of complementation of *rpt5a *mutation. Likewise, *rpt5a-4/RPT5a; RPT5b*^*Col-0*^/*RPT5b*^*Da-0 *^plants displayed 42% pollen lethality, while only 28% dead pollen was observed in a wild-type *RPT5a *background, which is consistent with an additive effect of the Col X Da-0 incompatibility described above and a *rpt5a-4; RPT5b*^*Da-0 *^male gametophyte lethality. Taken together, these data strongly suggest that the SNP2^Ws^, located in intron 7, is responsible for the lack of complementation of the *rpt5a *mutation by *RPT5b *loci.

### SNP2^Ws ^generates a new putative branchpoint consensus sequence in *RPT5b *intron 7

**Figure 3:**
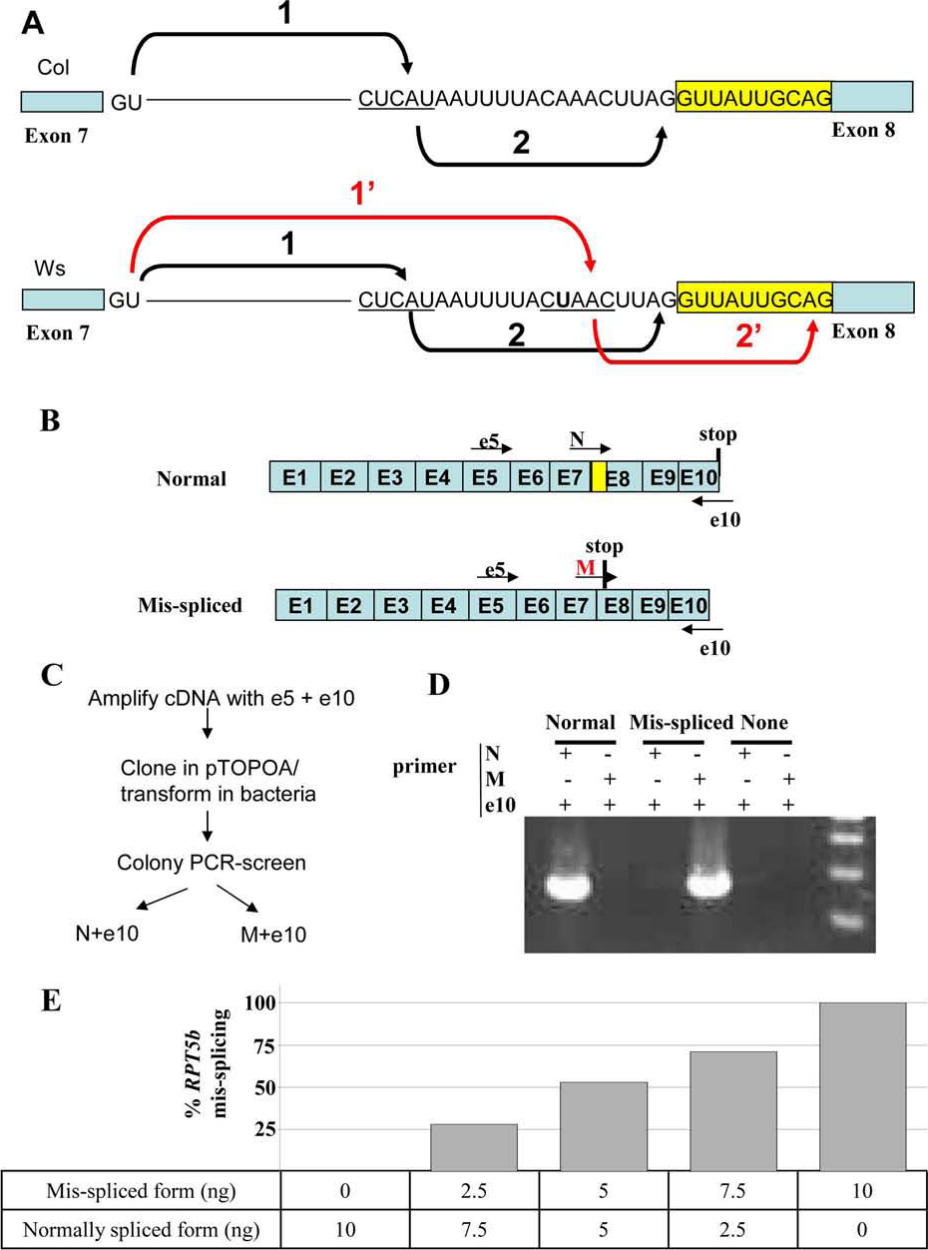
**Alternative splicing of *RPT5b*^*Ws*^**. **A**, Hypothesis for the cause of alternative splicing at intron seven of *RPT5b*. Schematic representation of the Col intron with the different stages of splicing depicted: formation of a lariat between the 5' splice site and the branchpoint (1) and cleavage of the 3' splice site (2) before ligation of the two exon sequences. In the Ws intron, the SNP2 variant (U, in bold character) generates a new branchpoint sequence that activates the selection of a downstream 3' splice site. Exons are represented by boxes. The yellow box corresponds to the beginning of exon 8 that is spliced out in Ws. **B**, Graphic representation of mis-splicing occurring at *RPT5b *intron 7. Primers used for the cDNA amplification and for the splicing characterisation are represented (See methods). **C**, Strategy used to assess the level of mis-splicing at intron 7. Normal spliced forms are amplified with primers N and e10 while the mis-spliced form is detected by amplification with primers M and e10, as exemplified in **D. E**, Plasmids containing both e5-e10 fragments of the normally spliced and mis-spliced form of *RTP5b *intron 7 were mixed up to 10 ng, as indicated below the graph. The level of mis-splicing was then assessed as represented in Fig. 3C. At least 100 cloned cDNAs were tested per genotype. Results show that our test gives a good estimate of the mis-spliced form *vs *total form ratio. Hence, in our conditions, there is no significant bias of PCR amplification and/or cloning of one spliced form over the other.

It had been previously shown that *RPT5b *is subject to alternative splicing at intron 7 [15]. The alternatively spliced mRNA carries a 10 bp deletion at the beginning of exon 8 and consequently a frameshift resulting in a stop codon at the beginning of exon 8, leading to a much shorter predicted protein (35 kDa instead of 47 kDa) (Fig. 3B). In plants, as in yeast and vertebrates, exons are excised by a two-step reaction (Fig. 3A): firstly the 5' splice site is cleaved and there is formation of a lariat with the branchpoint that is usually located 18 to 40 nts upstream of the 3' splice site. Then, in a second step, the 3' splice site is cleaved and both exons are ligated. The 3' splice site selection results from the spliceosome scanning for the first AG dinucleotide downstream of the branchpoint [16]. By analysing the intron 7 sequence in the Col-0 accession, one branchpoint sequence was found (CUCAU) that partially matches the plant consensus YURAY [17] with an A nucleotide located 18 bp upstream from the 3' splice site (Fig. 3A). By analysing the same intron sequence in the Ws accession, we found that SNP2^Ws ^generated a new putative branchpoint consensus (CUAAC) that with its upstream sequence perfectly matches the yeast highly conserved sequence (UACUAAC). Therefore, although additional experiments would be required to prove that this sequence is a *bona fide *branchpoint [17,18], this sequence is very likely to represent a branchpoint sequence that would compete with the upstream one. It has been shown that, particularly in mammalian genes, the 3' splicing site selection is defined by scanning for the first AG located 3' of the branchpoint [19], unless this AG is too close to the branchpoint (less than 12 nts), in which case steric effects would hamper the use of this AG in favour of a downstream AG [20]. In *RPT5b *alleles harbouring a SNP2^Ws ^signature, as the new branchpoint adenosine is located 6 nts from the 3' splice sequence, it is very likely that its use will trigger the spliceosome to scan until the next AG dinucleotide, leading to the mis-spliced RNA (Fig. 3B).

### SNP2^Ws ^correlates with *RPT5b *mis-splicing in the sporophyte

Previously, a test was developed to quantify the amount of *RPT5b *intron 7 mis-splicing among mRNA ([15] and Fig. 3C and 3D). Fragments of *RPT5b *cDNA surrounding intron 7 were cloned before analysis to make sure that the level of mis-splicing could be accurately assessed (Fig. 3E). However no significant difference could be detected in the level of *RPT5b *intron 7 mis-splicing between Col-0 and Ws-4 inflorescence [15]. One explanation would be that the mis-splicing occurring only in male gametophyte cells or mother cells would be diluted by splicing occurring in surrounding tissues, making it very difficult to analyse at this stage.

**Figure 4:**
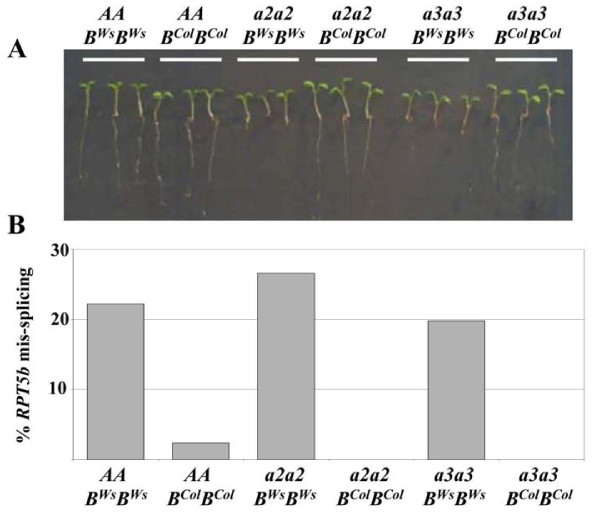
***RPT5b*^*Ws *^cDNA is partially mis-spliced, regardless of *RPT5a *status**. **A**, 6-day old seedlings grown on vertical agar plates from wild type *RPT5a *plants and *rpt5a2/rpt5a2 *and *rpt5a3/rpt5a3 *in either *RPT5b*^*Col-0*^/*RPT5b*^*Col-0 *^or *RPT5b*^*Ws-4*^/*RPT5b*^*Ws-4 *^background. **B**, Percentages of *RPT5b *intron 7 mis-splicing in the same plantlets as in **A**. At least 100 cloned cDNAs were tested per genotype.

For this reason we looked at young seedlings, as these represent another developmental phase where *RPT5a *and *RPT5b *unequal redundancy is detected. In the Ws-4 accession, no homozygous plants are recovered for the strong mutant allele *rpt5a-1*, because it is fully male gametophytic lethal. However, two weaker alleles, *rpt5a-2 *and *rpt5a-3*, were isolated that show some residual transmission through the male gametophyte [15] and for which some homozygous plants were recovered. Those plants, *rpt5a-2/rpt5a-2 *; *RPT5b^Ws-4^/RPT5b^Ws-4 ^*and *rpt5a-3/rpt5a-3 *; *RPT5b^Ws-4^/RPT5b^Ws-4^*, show reduced development and the seedlings exhibit short roots. This phenotype was reversed to wild-type by introgressing the *rpt5a-2 *(or *rpt5a-3*) mutation into a *RPT5b^Col-0^/RPT5b^Col-0 ^*background ([15] and Fig. 4A), proving that the dwarf phenotype resulted from unequal redundancy between *RPT5a *and *RPT5b^Ws-4^*. Therefore, *RPT5b *intron 7 splicing was assessed on six-day old *RPT5a/RPT5a*, *rpt5a-2/rpt5a-2 *and *rpt5a-3/rpt5a-3 *seedlings in either *RPT5b^Col-0^/RPT5b^Col-0 ^*or *RPT5b^Ws-4 ^*/*RPT5b^Ws-4 ^*background. A very clear correlation was observed between the presence of the *RPT5b^Ws-4 ^*allele and a ratio of 20 to 25% of mis-splicing among *RPT5b *RNA, leading to PTC (Fig. 4B).

**Figure 5:**
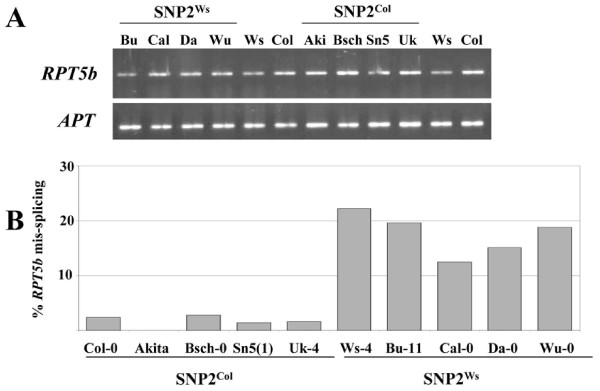
**Partial *RPT5b *mis-splicing correlates with *RPT5 *unequal redundancy**. **A**, RT-PCR on mRNA isolated from 6-day old seedlings from different accessions. *APT *was amplified as a control. **B**, Percentages of *RPT5b *intron 7 mis-splicing in the same plantlets as above. At least 100 cloned cDNAs were tested per genotype.

A similar study was carried out on all 8 accessions selected above as displaying a SNP1^Ws^/SNP2^Ws ^or SNP1^Ws^/SNP2^Col ^haplotype (Fig. 5A). A perfect correlation was observed between the presence of SNP2^Ws ^and a significant amount of mis-splicing at intron 7 (12 to 20%). Conversely, all 4 accessions with a SNP1^Col^/SNP2^Col ^haplotype showed a very limited amount or undetectable mis-splicing (0 to 2.8%) (Fig. 5B).

This demonstrates that SNP2^Ws^, located upstream of the 3'intron site consensus, is very likely responsible for a significant amount of mis-splicing that causes the lack of complementation of *rpt5a *mutation by *RPT5b *alleles carrying this SNP2^Ws^.

## Discussion

In the present study, we confirm that *RPT5a *and *RPT5b *display unequal redundancy in some *Arabidopsis *accessions and we characterized 4 new accessions in which the situation is similar. Moreover, by investigating *Arabidopsis *natural variation, we show that one SNP, located in *RPT5b *intron seven, causes this unequal redundancy between *RPT5a *and *RPT5b *genes. The A to T substitution in position -8 to the intron 3' splice site correlates with a 25% mis-splicing rate in Ws-4 and in 4 other accessions (Bu-11, Cal-0, Da-0 and Wu-0). Sequence analyses reveal that the SNP2^Ws ^generates a new putative branchpoint sequence that probably competes with the original branchpoint. As a result, another 3' splice site is selected downstream, resulting in a 10 bp deletion in the *RPT5b *mRNA. However, how this competition occurs between both branchpoint sequences, and consequently both 3' splice sites is unclear. In mammalian genes, despite the steric hindrance caused by the closeness between the branchpoint and the next downstream AG dinucleotide, AG sequences located as close as 4 nt downstream of the branchpoint would still be used at some extent [20]. Data suggest that 3' splicing site scanning occurs in plants as in mammals [17,21], explaining why even in the presence of SNP2^Ws^, the first AG remains the main 3' splicing site. In the 5 accessions carrying SNP2^Ws^, only a fraction of *RPT5b *pre-mRNA is mis-spliced (between 12 and 25%), though our assay might underestimate this percentage if some of the mis-spliced form is degraded through NMD, as it is frequently the case with mRNA displaying PTC [13,22]. However, semi-quantitative RT-PCR shows that *RPT5b *mRNAs accumulate similarly in all accessions we tested (Fig. 5A). The subtle defect in *RPT5b *pre-mRNA splicing may also explain why transgenes expressing various *RPT5b *constructs all complement a *rpt5a *strong male gametophyte defect: the additive expression of both the transgenic and endogenous *RPT5b *expression is probably sufficient to overcome the lack of RPT5a by providing enough functional RPT5b.

Presumably, this SNP2, through causing a "mild" effect on splicing, is likely to have a stronger effect on RPT5b protein accumulation with deleterious consequences on both male gametophyte and sporophyte development. Indeed, F2 plants that segregate with a *rpt5a-4/rpt5a-4; RPT5b^Col-0^/RPT5b^Bu-11 ^*genotype displayed a short root that is typical of mutants partially affected in the proteasome machinery such as *rpt2a*, *rpn10 *and *rpn12 *as well as weaker alleles of *rpt5a *[15,23,24] (Data not shown). This suggests that the unequal redundancy between *RPT5a *and the other *RPT5b *alleles bearing the SNP2^Ws ^extend to sporophyte development.

We previously pointed that while the lack of redundancy between *RPT5a *and *RPT5b^Ws-4 ^*has a dramatic effect on male gametophyte development (leading to pollen lethality) it has no or very little effect on the female gametophyte development [15]. Several studies point out that alternative splicing can be regulated by hormones, stresses or developmental cues (i.e. the pattern of alternative splicing can be organ-specific) [25-27]. In this regard, it is interesting to note a recently described set of mutants that impair female gametophyte development: *LACHESIS, CHLOTO/GAMETOPHYTIC FACTOR1 *and *ATROPOS *are essential for female gamete cell fate and all three genes encode spliceosomal components [28,29]. The authors speculate that splicing regulation could be required for maturing specific gametophytic factors mRNA. Likewise, it could be that male gametophytes also have a specific spliceosome machinery that would make them more susceptible to SNP2 ^Ws ^related-alternative splicing while the female machinery would not. It will be interesting to see whether mutations affecting male-gametophyte development will be discovered by screening for more mutations of the spliceosome machinery.

The *RPT5b *alternatively-spliced mRNA described here leads to PTC that is supposed to induce NMD [12], *i.e*. degradation of the mRNA to prevent the translation of an incomplete protein. Although alternative splicing has been proposed as a way to generate novel proteins and therefore enhance transcriptomic/proteomic plasticity, there are no indications that this is the case here: the present alternate splicing leads to a RPT5b devoid of the conserved AAA-ATPase domains H and K [30,31] that is likely to be a non-functional protein as is the C-term deleted RPT2a protein in the *hlr-2 *mutant [24]. Besides, PTC caused by alternative splicing has been demonstrated to act as a way to regulate gene function by the RUST mechanism (Regulated Unproductive Splicing and Translation) [32]: it triggers the production of mRNAs encoding non functional proteins and those mRNAs are the targets of NMD. However, we found that the level of *RPT5b^Ws-4 ^*mis-splicing was similar in seedlings in both wild-type *RPT5a *background and in *rpt5a-2 *or *rpt5a-3 *mutant background (Fig. 4B), suggesting that there is no feedback on *RPT5b *mis-splicing based on RPT5 protein content.

Alternative splicing has been extensively assessed in *Arabidopsis *[33] showing that up to 42% of intron-containing genes are alternatively spliced. The majority of those alternate transcripts lead to PTC, suggesting it has an important role for gene expression regulation. New sequencing methods as High Throughput Sequencing [33-35] offer the opportunity to assess how alternative splicing varies between accessions and to see how it can contribute to transcriptomic/proteomic diversification among natural variation.

## Conclusion

*RPT5a *and *RPT5b *both encode the RPT5 subunit, a component of the Regulatory Particle of the 26S proteasome, the complex that degrades ubiquitin-labelled targets as part of the Ubiquitin proteasome System (UPS) [36]. The 26S/UPS is an essential pathway involved in development and regulatory processes in eukaryotes, including plants [37,38]. In *Arabidopsis*, most of the 26S proteasome components are encoded by duplicated genes [39] and it is not clear whether these duplications are the basis of a mere redundancy or of a proteasome plasticity. In this regard, assessing the redundancy between *RPT5a *and *RPT5b *among natural variation in *Arabidopsis thaliana *brings new insights by showing that these genes offer another example of unequal redundancy [5] through alternative splicing in some unrelated accessions, without any apparent gain of functions. A recent study carried out in *Arabidopsis *shows that alternative splicing plays an important role for divergence between duplicated genes [26]. It would be interesting to test whether other proteasome subunits display such incompatibilities and therefore determine whether some of those other duplicated genes are drifting away or not.

## Methods

### Plant Materials and Growth Conditions

*Arabidopsis thaliana *Columbia (Col-0) and Wassilewskija (Ws-4) accessions were used as reference plants. The *rpt5a-4 *T-DNA insertion mutant allele has been already described [15]. It was isolated in the SALK collection as line S046321 in Col-0 accession (SIGnAL; http://signal.salk.edu/cgi-bin/tdnaexpress). As previously, the *rpt5a-4 *mutant allele was genotyped with the S46321-L and LBSalk2 primers while the wild-type allele was scored with S46321-U and S46321-L. All primers are described in Additional file 2: Table S1.

The 487-accessions collection (Additional file 3: Table S2.) was provided by the Resource Centre of INRA Versailles http://dbsgap.versailles.inra.fr/vnat. All Col (Col-0 and Col-7) and Ws accessions (Ws, Ws-1, Ws-2, Ws-3 and Ws-4) were found to present the same *RPT5b *haplotype; therefore only Col-0 and Ws-4 was used in this study. For growth on either plates or soil, seeds were stratified for 2 days at 4°C and grown at 18 to 20°C, with 16 h light (fluorescent lights: 100 μmol photons m^-2 ^s^-1^) and 8 h dark cycles. Immature flowers were emasculated and manually cross-pollinated for crosses.

### PCR-Genotyping of *RPT5b *SNPs

Genomic DNA of the 487 accessions was extracted in 96-well plates from seedlings, using a SDS-based buffer [40]. SNP1 and SNP2 were detected as previously reported using dCAPS markers [15]. For the SNP1 located in the *RPT5b *promoter, a 266 bp fragment was amplified with primers JLV096 and JLV097 and digested with *Vsp*I. Only the fragment originating from the Ws allele was restricted into 249 and 17 bp fragments. For the SNP2, located in the *RPT5b *intron seven, a 280 bp fragment was amplified with primers RPT5b-3369 and RPT5b-Hind3 and digested with *Hind*III. Only the fragment originating from the Col allele was restricted into 250 and 30 bp fragments.

### Histological Analysis

Pollen viability was assessed using Alexander staining [41] and observed with a PL Fluotar X25 dry objective on a Leitz Diaplan microscope.

### Expression Analysis

Total RNA was extracted using TRIZOL-reagent (Invitrogen) from 100 mg of tissue (6 day-old plantlets). Contaminating DNA was removed by DnaseI treatment with RNase Free DNase set (Qiagen) using spin columns of the Rneasy Plant mini kit (Qiagen).

RT-PCR was performed with RevertAid H Minus M-MuLV Reverse Transcriptase (Fermentas) on 500 ng RNA according to the supplier's instructions. *RPT5b *and *APT *cDNAs were amplified with specific primers: e5 and e10 for *RPT5b*, APT5' and APT3' for *APT*.

*RPT5b *e5-e10 cDNA fragments were amplified by 25 cycles of PCR with e5 and e10 primers. The PCR products were then cloned into pTOPOII plasmid (Invitrogen) and the resulting constructs transformed into DH10B competent cells. A hundred recombinant clones were analysed by PCR using N and e10 primers for normal cDNA detection and M and e10 primers for mis-spliced cDNA.

## Authors' contributions

AGD and JLG designed the experiments. AGD, AL and JLG carried out the experiments. AGD and JLG wrote the manuscript. SB and PG participated to the writing. All authors read and approved the final manuscript.

## Supplementary Material

Additional file 1 **Figure S1. **Transgenic *RPT5b *complements *rpt5a-1 *male transmission defect regardless of SNP1 and SNP2 genotypes. Click here for file

Additional file 2 **Table S1: List of primers used in this study**. Click here for file

Additional file 3 **Table S2: List of accessions screened in this study**. Click here for file

## References

[B1] Moore RC Purugganan MD The evolutionary dynamics of plant duplicate genes Curr Opin Plant Biol 2005 8 2 122 128 10.1016/j.pbi.2004.12.001 15752990

[B2] Bowers JE Chapman BA Rong J Paterson AH Unravelling angiosperm genome evolution by phylogenetic analysis of chromosomal duplication events Nature 2003 422 6930 433 438 10.1038/nature01521 12660784

[B3] Blanc G Hokamp K Wolfe KH A recent polyploidy superimposed on older large-scale duplications in the Arabidopsis genome Genome Res 2003 13 2 137 144 10.1101/gr.751803 12566392 PMC420368

[B4] Ohno S Evolution by Gene Duplication 1970 London: George Allen and Unwin

[B5] Briggs GC Osmont KS Shindo C Sibout R Hardtke CS Unequal genetic redundancies in Arabidopsis--a neglected phenomenon? Trends Plant Sci 2006 11 10 492 498 10.1016/j.tplants.2006.08.005 16949326

[B6] Bikard D Patel D Le Mette C Giorgi V Camilleri C Bennett MJ Loudet O Divergent evolution of duplicate genes leads to genetic incompatibilities within A. thaliana Science 2009 323 5914 623 626 10.1126/science.1165917 19179528

[B7] Bomblies K Lempe J Epple P Warthmann N Lanz C Dangl JL Weigel D Autoimmune response as a mechanism for a Dobzhansky-Muller-type incompatibility syndrome in plants PLoS Biol 2007 5 9 e236 10.1371/journal.pbio.0050236 17803357 PMC1964774

[B8] Alonso-Blanco C Aarts MG Bentsink L Keurentjes JJ Reymond M Vreugdenhil D Koornneef M What has natural variation taught us about plant development, physiology, and adaptation? Plant Cell 2009 21 7 1877 1896 10.1105/tpc.109.068114 19574434 PMC2729614

[B9] Werner JD Borevitz JO Uhlenhaut NH Ecker JR Chory J Weigel D FRIGIDA-independent variation in flowering time of natural Arabidopsis thaliana accessions Genetics 2005 170 3 1197 1207 10.1534/genetics.104.036533 15911588 PMC1451178

[B10] Yuan YX Wu J Sun RF Zhang XW Xu DH Bonnema G Wang XW A naturally occurring splicing site mutation in the Brassica rapa FLC1 gene is associated with variation in flowering time J Exp Bot 2009 60 4 1299 1308 10.1093/jxb/erp010 19190098 PMC2657548

[B11] Tarrio R Ayala FJ Rodriguez-Trelles F Alternative splicing: a missing piece in the puzzle of intron gain Proc Natl Acad Sci USA 2008 105 20 7223 7228 10.1073/pnas.0802941105 18463286 PMC2438231

[B12] Barbazuk WB Fu Y McGinnis KM Genome-wide analyses of alternative splicing in plants: opportunities and challenges Genome Res 2008 18 9 1381 1392 10.1101/gr.053678.106 18669480

[B13] Maquat LE Nonsense-mediated mRNA decay: splicing, translation and mRNP dynamics Nat Rev Mol Cell Biol 2004 5 2 89 99 10.1038/nrm1310 15040442

[B14] Wang BB Brendel V Genomewide comparative analysis of alternative splicing in plants Proc Natl Acad Sci USA 2006 103 18 7175 7180 10.1073/pnas.0602039103 16632598 PMC1459036

[B15] Gallois JL Guyon-Debast A Lecureuil A Vezon D Carpentier V Bonhomme S Guerche P The Arabidopsis proteasome RPT5 subunits are essential for gametophyte development and show accession-dependent redundancy Plant Cell 2009 21 2 442 459 10.1105/tpc.108.062372 19223514 PMC2660631

[B16] Brown JW Simpson CG Splice site selection in plant pre-mRNA splicing Annu Rev Plant Physiol Plant Mol Biol 1998 49 77 95 10.1146/annurev.arplant.49.1.77 15012228

[B17] Simpson CG Clark G Davidson D Smith P Brown JW Mutation of putative branchpoint consensus sequences in plant introns reduces splicing efficiency Plant J 1996 9 3 369 380 10.1046/j.1365-313X.1996.09030369.x 8919913

[B18] Liu HX Filipowicz W Mapping of branchpoint nucleotides in mutant pre-mRNAs expressed in plant cells Plant J 1996 9 3 381 389 10.1046/j.1365-313X.1996.09030381.x 8919914

[B19] Smith CW Porro EB Patton JG Nadal-Ginard B Scanning from an independently specified branch point defines the 3' splice site of mammalian introns Nature 1989 342 6247 243 247 10.1038/342243a0 2812024

[B20] Smith CW Chu TT Nadal-Ginard B Scanning and competition between AGs are involved in 3' splice site selection in mammalian introns Mol Cell Biol 1993 13 8 4939 4952 8336728 10.1128/mcb.13.8.4939-4952.1993 PMC360135

[B21] Tanaka A Mita S Ohta S Kyozuka J Shimamoto K Nakamura K Enhancement of foreign gene expression by a dicot intron in rice but not in tobacco is correlated with an increased level of mRNA and an efficient splicing of the intron Nucleic Acids Res 1990 18 23 6767 6770 10.1093/nar/18.23.6767 2263444 PMC332729

[B22] Palusa SG Reddy AS Extensive coupling of alternative splicing of pre-mRNAs of serine/arginine (SR) genes with nonsense-mediated decay New Phytol 185 1 83 89 10.1111/j.1469-8137.2009.03065.x 19863731

[B23] Kurepa J Toh EA Smalle JA 26S proteasome regulatory particle mutants have increased oxidative stress tolerance Plant J 2008 53 1 102 114 10.1111/j.1365-313X.2007.03322.x 17971041

[B24] Ueda M Matsui K Ishiguro S Sano R Wada T Paponov I Palme K Okada K The HALTED ROOT gene encoding the 26S proteasome subunit RPT2a is essential for the maintenance of Arabidopsis meristems Development 2004 131 9 2101 2111 10.1242/dev.01096 15073153

[B25] Palusa SG Ali GS Reddy AS Alternative splicing of pre-mRNAs of Arabidopsis serine/arginine-rich proteins: regulation by hormones and stresses Plant J 2007 49 6 1091 1107 10.1111/j.1365-313X.2006.03020.x 17319848

[B26] Zhang PG Huang SZ Pin AL Adams KL Extensive Divergence in Alternative Splicing Patterns After Gene and Genome Duplication During the Evolutionary History of Arabidopsis Mol Biol Evol 20185454 10.1093/molbev/msq054

[B27] Simpson CG Fuller J Maronova M Kalyna M Davidson D McNicol J Barta A Brown JW Monitoring changes in alternative precursor messenger RNA splicing in multiple gene transcripts Plant J 2008 53 6 1035 1048 10.1111/j.1365-313X.2007.03392.x 18088312

[B28] Moll C von Lyncker L Zimmermann S Kagi C Baumann N Twell D Grossniklaus U Gross-Hardt R CLO/GFA1 and ATO are novel regulators of gametic cell fate in plants Plant J 2008 56 6 913 921 10.1111/j.1365-313X.2008.03650.x 18702672

[B29] Gross-Hardt R Kagi C Baumann N Moore JM Baskar R Gagliano WB Jurgens G Grossniklaus U LACHESIS restricts gametic cell fate in the female gametophyte of Arabidopsis PLoS Biol 2007 5 3 e47 10.1371/journal.pbio.0050047 17326723 PMC1804285

[B30] Fu H Doelling JH Rubin DM Vierstra RD Structural and functional analysis of the six regulatory particle triple-A ATPase subunits from the Arabidopsis 26S proteasome Plant J 1999 18 5 529 539 10.1046/j.1365-313X.1999.00479.x 10417703

[B31] Beyer A Sequence analysis of the AAA protein family Protein Sci 1997 6 10 2043 2058 10.1002/pro.5560061001 9336829 PMC2143574

[B32] Lareau LF Green RE Bhatnagar RS Brenner SE The evolving roles of alternative splicing Curr Opin Struct Biol 2004 14 3 273 282 10.1016/j.sbi.2004.05.002 15193306

[B33] Filichkin SA Priest HD Givan SA Shen R Bryant DW Fox SE Wong WK Mockler TC Genome-wide mapping of alternative splicing in Arabidopsis thaliana Genome Res 2010 20 1 45 58 10.1101/gr.093302.109 19858364 PMC2798830

[B34] Pickrell JK Marioni JC Pai AA Degner JF Engelhardt BE Nkadori E Veyrieras JB Stephens M Gilad Y Pritchard JK Understanding mechanisms underlying human gene expression variation with RNA sequencing Nature 2010 464 7289 768 772 10.1038/nature08872 20220758 PMC3089435

[B35] Shendure J Ji H Next-generation DNA sequencing Nat Biotechnol 2008 26 10 1135 1145 10.1038/nbt1486 18846087

[B36] Smalle J Vierstra RD The ubiquitin 26S proteasome proteolytic pathway Annu Rev Plant Biol 2004 55 555 590 10.1146/annurev.arplant.55.031903.141801 15377232

[B37] Vierstra RD The ubiquitin-26S proteasome system at the nexus of plant biology Nat Rev Mol Cell Biol 2009 10 6 385 397 10.1038/nrm2688 19424292

[B38] Hershko A Ciechanover A The ubiquitin system Annu Rev Biochem 1998 67 425 479 10.1146/annurev.biochem.67.1.425 9759494

[B39] Kurepa J Smalle JA Structure, function and regulation of plant proteasomes Biochimie 2008 90 2 324 335 10.1016/j.biochi.2007.07.019 17825468

[B40] Simon M Loudet O Durand S Berard A Brunel D Sennesal FX Durand-Tardif M Pelletier G Camilleri C Quantitative trait loci mapping in five new large recombinant inbred line populations of Arabidopsis thaliana genotyped with consensus single-nucleotide polymorphism markers Genetics 2008 178 4 2253 2264 10.1534/genetics.107.083899 18430947 PMC2323813

[B41] Alexander MP Differential staining of aborted and nonaborted pollen Stain Technol 1969 44 3 117 122 4181665 10.3109/10520296909063335

