# Supplementary figures and images for: A SNP associated with alternative splicing of RPT5b causes unequal redundancy between RPT5a and RPT5b among Arabidopsis thaliana natural variation
Source: BMC Plant Biol. 2010 Aug 3;10:158. doi: 10.1186/1471-2229-10-158 (PMC3017782; doi:10.1186/1471-2229-10-158)

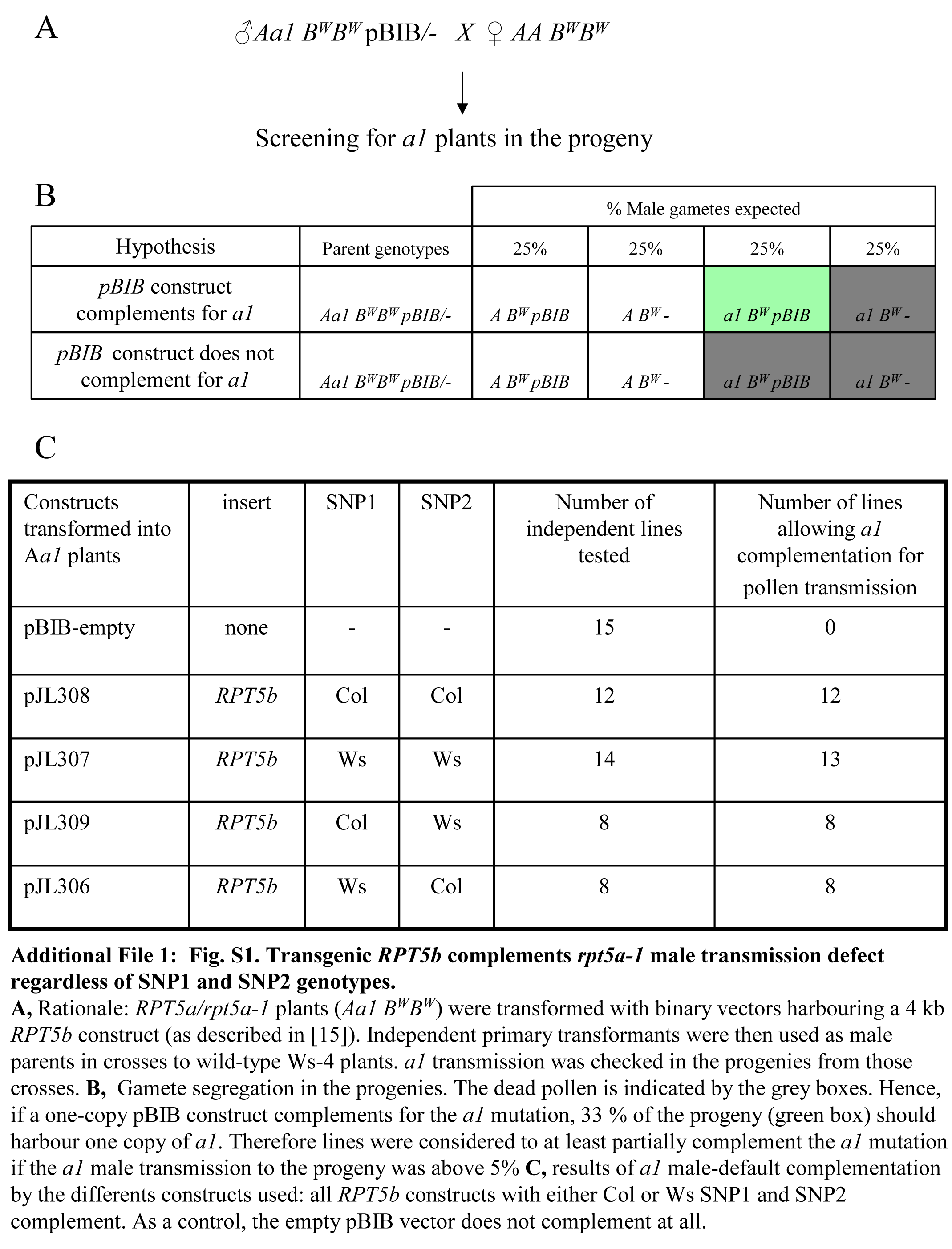

Supplement: Additional file 1 — Figure S1. Transgenic RPT5b complements rpt5a-1 male transmission defect regardless of SNP1 and SNP2 genotypes. [file 1471-2229-10-158-S1.TIFF]
